# Oxidative Stress after Surgery on the Immature Heart

**DOI:** 10.1155/2016/1971452

**Published:** 2016-03-31

**Authors:** Daniel Fudulu, Gianni Angelini

**Affiliations:** Bristol Heart Institute, Level 7, Upper Maudlin Street, Bristol BS2 8HW, UK

## Abstract

Paediatric heart surgery is associated with increased inflammation and the production of reactive oxygen species. Use of the extracorporeal cardiopulmonary bypass during correction of congenital heart defects generates reactive oxygen species by various mechanisms: haemolysis, neutrophil activation, ischaemia reperfusion injury, reoxygenation injury, or depletion of the endogenous antioxidants. The immature myocardium is more vulnerable to reactive oxygen species because of developmental differences compared to the adult heart but also because of associated congenital heart diseases that can deplete its antioxidant reserve. Oxidative stress can be manipulated by various interventions: exogenous antioxidants, use of steroids, cardioplegia, blood prime strategies, or miniaturisation of the cardiopulmonary bypass circuit. However, it is unclear if modulation of the redox pathways can alter clinical outcomes. Further studies powered to look at clinical outcomes are needed to define the role of oxidative stress in paediatric patients.

## 1. Introduction

The stress response to surgery compromises a series of humoral, metabolic, or cellular reactions [1]. Cardiac surgery with use of cardiopulmonary bypass (CPB) is a major activator of the systemic inflammatory response (SIRS) [2, 3]. In some instances, SIRS, rather than being a homeostatic mechanism, can be overactivated and result in multiorgan failure and increased mortality after surgery [2]. Inflammation, resulting in neutrophil activation, plays a central role in the production of reactive oxygen species (ROS) [4, 5]. However, other pathways such as haemolysis, ischaemic reperfusion injury, or reoxygenation of the hypoxic myocardium can also generate free radicals. When there is an imbalance between the production of ROS and the antioxidant capacity of the body,* oxidative stress* occurs resulting in cellular injury. Very few studies looked at the impact of oxidative stress on the immature heart, and little is known about the differences between the developing heart and the mature heart during oxidative stress. Although the immature heart has a greater antioxidant capacity than the adult heart, certain congenital conditions that we address surgically make it more susceptible to free radical oxidation [6]. Theoretically, modulation of these oxidative pathways could be of great importance to clinical practice. However, it is unclear how oxidative stress correlates with clinical outcomes after paediatric heart surgery. The current review focuses on the mechanisms of free radical production during paediatric heart surgery, the particularities that make the immature heart more prone to oxidative damage but also on the possible interventions that could mitigate ROS production.

## 2. Free Radicals, Reactive Oxygen Species, Mitochondrial Oxidative Stress, and Antioxidant Enzymes

*Free radicals* are chemical species with a single unpaired electron making them highly unstable and reactive. The role of oxygen derived free radicals was studied in the context of cell injury secondary to ischaemic reperfusion injury, inflammation, phagocytosis, chemical or radiation injury, oxygen toxicity, and cell aging [7]. Reactive oxygen species refers to free radicals and other oxidants without an unpaired electron such as the highly reactive hydrogen peroxide (H_2_O_2_).

The main types of reactive oxygen species are the superoxide anion (O_2_^∙−^), hydrogen peroxide (H_2_O_2_), the hydroxyl radicals (^*∙*^OH), peroxynitrite (ONOO^−^), and the hypochlorite radical (OCl^−^) [2, 5–8].

Reactive oxygen species are produced under physiological conditions during the mitochondrial respiration process by the reduction-oxidation reactions (redox). A toxic reactant escaping this reaction is the O_2_^∙−^ radical that can convert, spontaneously or under the action of the superoxide dismutase, to H_2_O_2_. The so-called* Fenton reaction* can further generate from H_2_O_2_ the highly reactive hydroxyl radical (^*∙*^OH).

Mitochondria are the energy factory of the body but also a major source of reactive oxygen. The mitochondrial transport chain can leak electrons to molecular oxygen resulting in superoxide radical production. When the mitochondrial antioxidant capacity is overwhelmed,* mitochondrial oxidative stress* can cause mitochondrial DNA damage, impaired mitochondrial respiration, and lipid or protein peroxidation. Such processes are responsible for DNA mutations, cellular aging, and death. An important mechanism is the peroxidation of* cardiolipin*, a mitochondrial lipid protein that results in cytochrome C release and subsequent caspase activation and apoptosis [9–13].

A recently discovered antioxidant system is the mitochondrial* thioredoxin* and* peroxiredoxin* family of proteins [14]. This scavenger complex is an important regulator of the redox state but also a regulator of cell apoptosis via the apoptosis stress kinase 1 (ASK 1). The mitochondrial thioredoxin-2 can preserve cardiac function by suppressing ROS within the mitochondria according to a study [15].

Within the activated phagocyte, a similar chain of reactions occurs. Of great importance is the production of the superoxide anion (O_2_^∙−^) by the reaction of oxygen with nicotinamide adenine dinucleotide phosphate (NADPH). This reaction is catalysed by the NADPH-oxidase, a membrane specific enzyme. Another key enzyme is the* myeloperoxidase* that catalyses the reaction of the halide ions (Cl^−^) with H_2_O_2_, thus generating the OCl^−^, the “bleach” of the phagocyte [5].

**Figure 1:**
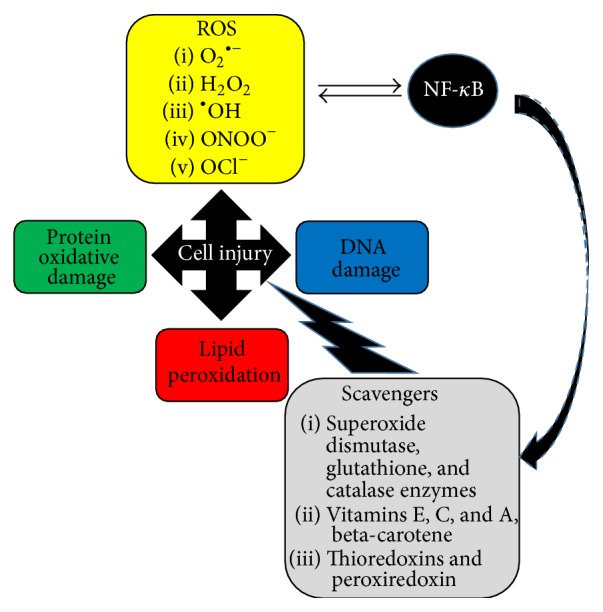
The various reactive oxygen species (ROS) injure cells by three main mechanisms: lipid peroxidation, protein oxidative damage, and DNA damage. Cellular injury is limited by the action of the main antioxidant enzymes (scavengers). There is a complex cross talk between ROS and NF-*κ*B transcription factors that regulate the production of antioxidants.

Cell injury occurs by three main mechanisms: lipid peroxidation, protein oxidative damage, and DNA damage (Figure 1) [7, 8]. Nonspecific markers of lipid peroxidation such as the thiobarbituric acid-reactive substances (TBARS) had been used in previous studies to measure outcomes after paediatric heart surgery [16]. Antioxidant enzymes such as the* superoxide dismutase*, the* glutathione* synthetic enzymes, and the* catalases* can protect cells against the oxidative stress. As we will see later, both endogenous and exogenous antioxidants such as vitamins C, E, and A or beta-carotene can also enhance the antioxidant capacity [7].

Apart from their important role in microbial killing, ROS are also involved in cell signaling [7, 17, 18]. The NF-*κ*B family of transcription factors are involved in inflammation, immunity, cellular growth, or apoptosis. There is a complex cross talk between ROS and NF-*κ*B: transcription of the NF-*κ*B gene that regulates the production of ROS in the cell but also NF-*κ*B activity can be influenced by ROS. Furthermore, NF-*κ*B can mediate the expression of antioxidant proteins such as superoxide dismutases, ferritin heavy chain, thioredoxins, and glutathione peroxidase [18].

## 3. Paediatric Extracorporeal Circuits and Oxidative Stress

Use of the extracorporeal circuits induces oxidative stress in various ways, ultimately resulting in organ system dysfunction (Figure 1).

### 3.1. Extracorporeal Circuits, Inflammation, Oxidative Stress, and Markers of Oxidation

Several factors cause a more profound systemic inflammatory response in neonates and infants compared to older children or adults: (1) the surface and the volume of the CPB circuit relative to the blood volume and patient size, (2) more frequent use of hypothermic circulatory arrest, and (3) more pronounced haemodilution [6]. It is well known that CPB induces systemic inflammation by mechanisms such as contact activation with the non-self-circuit surfaces, translocation of intestinal endotoxins, general surgical trauma, blood loss, or hypothermia [2, 19]. This activates the complement system, the production of cytokines, neutrophil adhesion and aggregation, nicotinamide adenine dinucleotide phosphate (NADPH) oxidase, and xanthine oxidase systems, ultimately resulting in ROS production [2, 4, 5, 8, 20]. Certainly, the recruitment and activation of neutrophils play a pivotal role in the production of ROS. Activation of neutrophils leads to an increase of the plasma neutrophil elastase and the increase of* myeloperoxidase* enzyme linked to oxidative stress [21]. Measurement of the myeloperoxidase can be used as a marker of cardiovascular disease [22].

Calza et al. [23] evaluated the glutathione redox cycle in the context of paediatric CPB. They found increased free radical production before and during CPB by measuring an increase of the total and oxidised glutathione (reduction of the glutathione/glutathione oxidase ratio).

Gil-Gómez et al. [24] found a direct correlation between the time of extracorporeal circulation, the duration of postoperative mechanical ventilation, and the amplitude of the oxidative stress response. The authors measured glutathione levels but also the lipid peroxidation product, malondialdehyde. Other products of lipid peroxidation such as isoflurane or 8-isoprostane were found to be increased during paediatric CPB [23, 25, 26].

Extracorporeal membrane oxygenation (ECMO) is commonly used in paediatric heart surgery. Similar to the CPB circuit, contact activation initiates an inflammatory cascade and oxidative stress. Extracorporeal membrane oxygenation is a major activator of SIRS because the whole blood of the patient is in contact for days or weeks with the ECMO circuit [8]. Hirthler et al. [27] in the nonsurvivors after paediatric ECMO found elevated levels of the lipid peroxidation markers: thiobarbiturate acid-reactive substances (TBARS) and malondialdehyde.

### 3.2. Ischaemic Reperfusion Injury and Oxidative Stress in the Immature Myocardium

**Figure 2:**
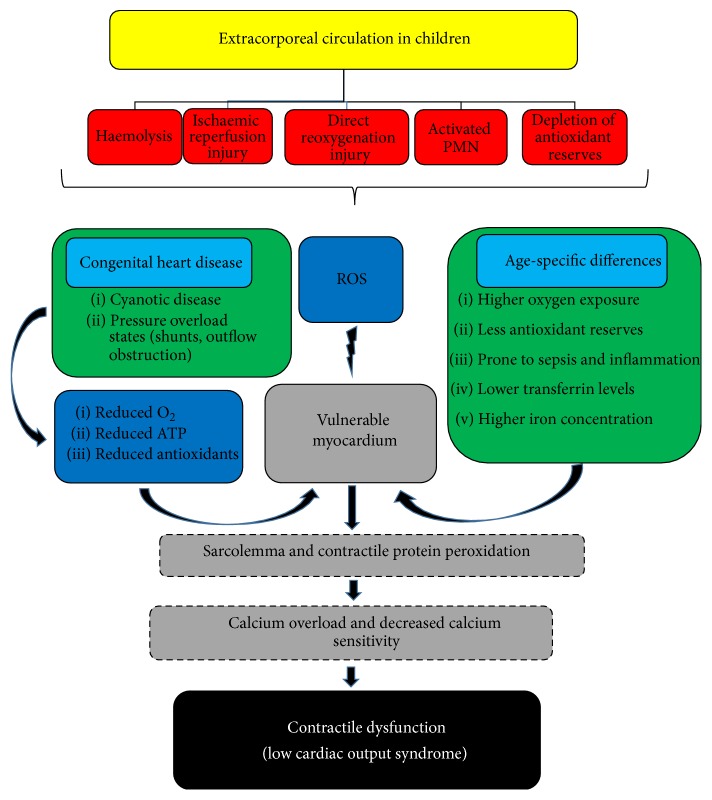
The various mechanisms of production of ROS with use of extracorporeal circuit. The immature myocardium is vulnerable to ROS injury because of age specific differences compared to the adult heart but also because of coexistent congenital heart disease. The end-result is contractile dysfunction.

Ischaemic reperfusion injury (IRI) following cardioplegic arrest results in increased ROS production. The main sources of ROS during IRI are (1) uncoupling of the mitochondrial electron transport, (2) circulating polymorphonuclear leukocytes generating superoxide anions from NADPH (reaction catalysed by the NAPDH oxidase), and (3) coronary endothelial cells that generate the superoxide anion from hypoxanthine, reaction catalysed by the* xanthine oxidase* enzyme [28]. Ischaemic reperfusion injury results in cardiac dysfunction, a major cause of mortality and morbidity in paediatric cardiac surgery [16, 29, 30]. Reactive oxygen species can induce contractile dysfunction (low cardiac output syndrome) by peroxidation of the sarcolemma and of the contractile proteins. This results in calcium overload and calcium desensitization (Figure 2) [28].

Previous studies suggested that the immature heart is more tolerant to ischaemia than the adult heart. Possible mechanisms implicated are as follows: (1) the sarcolemma of the cell is more resistant to calcium, (2) the immature myocardium relies on fatty acid for energy production and hence less potential for anaerobic glycolysis, and (3) the larger amount of amino acids provides more substrates of anaerobic metabolism [6, 16, 23]. Despite such developmental differences, the myocardium in cyanotic heart disease and heart failure might be more susceptible to ischaemia and subsequently oxidative stress [16, 23, 30]. The pressure overload of the myocardium in left to right shunts or outflow obstructions, or the reduced oxygen availability in cyanotic heart disease, further reduces the ATP stores and antioxidant enzymes, thus making the myocardium more susceptible to oxidative injury [23, 31, 32]. Cabigas et al. [33] compared oxidative stress in the newborn and adult heart in a model of ischaemic reperfusion injury. They found age and chamber specific differences related to oxidative stress. Interestingly, the newborn right ventricle myocytes showed significantly higher production of H_2_O_2_ compared to the adult heart, the superoxide dismutase activity increased only in the right ventricle heart, and the catalase activity and levels were all reduced in the newborn heart.

Reactive oxygen species play a fundamental role in reperfusion injury and their effect had been extensively studied in this context [8]. During IRI, oxidants are produced by various mechanisms such as increased production of xanthine oxidase production by the endothelial cells, decreased function of the glutathione peroxidase enzyme, or leakage of electrons from the mitochondria (resulting in superoxide anions) [8]. The activated neutrophil with subsequent fabrication of ROS seems to play a role in more prolonged periods of ischaemia that are associated with tissue necrosis [8, 34]. Oliveira et al. [35] in an immunohistological study of infants that underwent repair of cardiac malformations found lipid peroxidation to be the mechanism responsible for myocardial injury. The authors tested immunoreactivity to 4-hydroxynonenal, a lipid peroxidation product, and nitrotyrosine, a tyrosine nitration product, mediated by the peroxynitrite radical.

Manso et al. [16] questioned the role of oxidative stress in myocardial dysfunction or low cardiac output after heart surgery. The authors found no correlation between TBARS and carbonyl moieties and the development of low cardiac output syndrome in a retrospective study of 55 children. Certainly, further studies powered to look at clinical outcomes and oxidative stress in paediatric surgery are required.

### 3.3. Hypoxemic/Reoxygenation Induced Oxidative Stress in Cyanotic Heart Disease

Buckberg et al. [29, 36] demonstrated in animal studies that the reintroduction of oxygen in the initial stages of CPB or mechanical ventilation can induce* per se* myocardial injury in the hypoxaemic myocardium. In cyanotic heart disease, the reduction of the antioxidant reserve increases the vulnerability of the myocardium. The authors demonstrated that reoxygenation reduced the myocardial reserve capacity (measured by incubating myocardial tissue in the oxidant t-butyl hydroperoxide) and increased the products of lipid peroxidation (conjugated dienes) measured in coronary sinus and the myocardial tissue.

Caputo et al. randomized cyanotic patients to receive either normoxic or hyperoxic cardiopulmonary bypass. The normoxic arm of patients had less oxidative stress (significantly lower 8-isoprostane levels) compared to the hyperoxic group [26]. Later studies by the same group demonstrated less oxidative stress with controlled reoxygenation in the single ventricle compared to double ventricle patients [25].

### 3.4. Extracorporeal Circuits and the Antioxidant Reserve

Oxidative stress during CPB is an imbalance between the production of free radicals and the antioxidant capacity of the body. As discussed earlier, congenital cardiac conditions are associated with a decrease of the myocardial antioxidant capacity. However, CPB can also deplete the plasma antioxidant capacity. Cavarocchi et al. demonstrated depletion of vitamin E after bypass [37, 38]. Pyles et al. [39] investigated in vitro the plasma ability to prevent lipid peroxidation (malondialdehyde production) in beef brain homogenate media. They found the plasma antioxidant capacity significantly reduced after congenital heart surgery with CPB.

### 3.5. Haemolysis, Blood Transfusion, and Iron Overload Promote Oxidative Stress 

The contact with the nonphysiological surfaces of the bypass circuit and the associated mechanical shear stress associated with CPB leads not only to inflammation but also to haemolysis [40–42]. The resulting free haemoglobin can react with H_2_O_2_ and generate redox active low molecular mass iron that can lead to lipid peroxidation and the highly reactive hydroxyl radical (^*∙*^OH) [42]. Iron overload in paediatric cardiac surgery can result also from blood cardioplegia, ischaemia reperfusion injury, blood transfusion, or the use blood prime [40].

Blood transfusion is a common practice in heart surgery that promotes oxidative stress by both the decreased antioxidant properties of stored blood and increased erythrocyte fragility resulting in haemolysis and ROS generation [4]. Low molecular mass iron is present in small amounts within cells for synthesis of fetoproteins or DNA. Under normal conditions, iron is regulated by ligands such as transferrin that inhibits transfer of electron from iron to molecular oxygen [42, 43]. When the iron binding capacity of transferrin is exceeded, free iron can be detected in the plasma. Notably, Mumby et al. [40] found a higher plasma iron overload in neonates undergoing CPB compared to older children. A possible explanation was the lower transferrin concentration within this group. Another study of patients undergoing tetralogy of Fallot repair found acute right ventricular failure due to restrictive physiology to be associated with severe iron loading of transferrin and increased oxidative stress markers compared to the nonrestrictive physiology cohort [44]. Two paediatric studies [42, 45] found associations between haemolysis and renal dysfunction that was believed to be mediated by lipid peroxidation.

Christen et al. [19] found the peak of oxidative stress markers (malondialdehyde and carbonyls) to occur before the rise of the inflammatory cytokines (interleukin-6 and interleukin-8) in paediatric patients undergoing heart surgery with CPB. This further suggests that mechanisms such as haemolysis could be responsible for the propagation of oxidative stress.

## 4. Modulation of Oxidative Stress in Paediatric Heart Surgery

**Figure 3:**
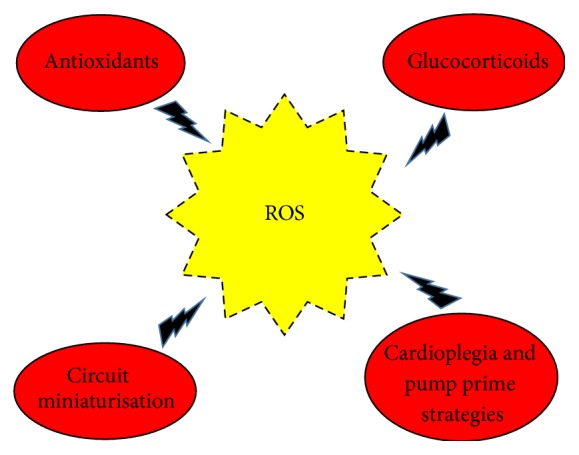
Interventions to reduce oxidative stress in paediatric heart surgery.

Figure 3 summarises the various interventions on oxidative stress after heart surgery.

### 4.1. Antioxidants

Children, particularly newborns, are more prone to oxidative stress because of several factors: (1) surfactant deficiency that exposes them to higher oxygen concentration, (2) less efficient antioxidant reserves, (3) being more prone to sepsis and inflammation, and (4) higher levels of free iron than older children [14]. Buckberg [29] studies defined the concept of antioxidant reserve capacity on the immature heart by incubating cardiac muscle with oxidants and measuring the products of lipid peroxidation. The dose response was similar to the Starling volume load curves and suggested the relation between antioxidant depletion and oxidative stress. Cardiopulmonary bypass had been shown to reduce plasma antioxidant capacity [19, 39]. Christen et al. [19] found a decrease in ascorbate (vitamin C) levels coupled with an increase in dehydroascorbate (oxidised vitamin C) and malondialdehyde levels in paediatric patients undergoing heart surgery. Therefore, increasing the antioxidant reserve capacity by administration of exogenous agents seems beneficial.

As seen previously, iron can play an important role in free radical formation; hence, chelators such deferoxamine could prove to be advantageous. Morita et al. [32] found administration deferoxamine to increase the heart antioxidant reserve in piglets undergoing CPB.

Data from critically ill patients or adult patients undergoing heart surgery suggests that supplementation of antioxidant micronutrients such as vitamin C, selenium, zinc, or vitamin E could increase the antioxidant defence [2, 4, 8, 46]. Amino acids are well known to have antioxidants properties [4]. For example, L-arginine reduced markers of oxidative stress in adult cardiac surgery when added to the cardioplegia solution [47]. The nonessential amino acid glutamine is another important endogenous antioxidant that could be supplemented [8]. N-acetylcysteine, antioxidant, and mucolytic agent used in respiratory medicine reduced the incidence of postoperative atrial fibrillation in a randomized controlled trial [48]; however, a later meta-analysis of randomized trials showed no benefit in reducing renal dysfunction, haemodialysis, or death [49]. To our knowledge, there are no studies investigating the effect of the above agents on oxidative stress in children undergoing heart surgery.

England et al. [50] found mannitol to reduce lipid peroxidation in patients undergoing adult heart surgery; however, its use was not evaluated in paediatric heart surgery. Allopurinol, an inhibitor of the purine metabolism, acts on the xanthine oxidase pathway generation [50] of superoxide. Some studies suggested reduced oxidative stress in paediatric heart surgery [50, 51] while others suggested it might not be beneficial [52]. One study on neonates with respiratory failure requiring ECMO demonstrated reduced purine degradation and uric acid production, hence with possible reduction in free radicals during reperfusion and reoxygenation injury [53].

The induction and maintenance anaesthetic agent, propofol, exerts antioxidant effects on the adult heart [4]. Xia et al. [54] in a study on 20 children undergoing CPB demonstrated reduced expression of NF-*κ*B in the propofol group. As seen earlier, oxidative stress is associated with the activation of the NF-*κ*B transcriptional factor. The same research group compared the antioxidant effects of the sedative midazolam to those of propofol in children undergoing congenital heart surgery. The propofol group had significantly less oxidative stress (lower superoxide dismutase and malondialdehyde levels) compared to the midazolam group [55].

*Salvia miltiorrhiza*, a herb extract with potent antioxidant effects, prevented the increase of lipid peroxidation products (malondialdehyde) in children undergoing CPB according to a study [56].

Melatonin is an endogenous indolamine, known to be a potent anti-inflammatory molecule but also a direct antioxidant [46, 57, 58]. In neonatal sepsis [46] but also after neonatal gastrointestinal surgery [57], melatonin reduced markers of oxidative stress. In several studies, using human cardiomyocytes cultures or isolated perfused rat heart, melatonin effectively reduced oxidative damage [59]. Further clinical studies in paediatric heart surgery are required to validate the above findings.

Aprotinin is used in congenital heart surgery as haemostatic agent but is also known to exert anti-inflammatory effects. Several paediatric studies suggested reduced oxidative stress with its use [60, 61].

As seen earlier, the mitochondrion is the main contributor to ROS production. Recently, agents that target the mitochondrial redox systems are being developed. MitoQ is a ubiquinone derivate conjugated to triphenylphosphonium that accumulates within the mitochondria because of an electrochemical gradient [4]. Ubiquinone is known for inhibiting the production of lipid peroxyl in the cell [13]. MitoQ reduced IRI in murine models of heart infarction [62] or heart transplant [12]. As opposed to the MitoQ lipophilic antioxidant that accumulates within the mitochondria in a potential-dependent manner, a novel class of small peptides (Szeto-Schiller) that selectively permeate the mitochondrial membrane had also been developed [10]. Szeto-Schiller peptides reduced IRI in several in vivo and ex vivo experimental models [10].

### 4.2. Glucocorticoids and CPB-Induced Oxidative Stress

Glucocorticoids are widely used in paediatric heart surgery to blunt the systemic inflammatory response to surgery but also to treat presumed postoperative adrenal insufficiency. Checchia et al. [63] in an international survey on steroid use in paediatric heart surgery found that 35 out of 36 centres (97%) give steroids but there is very wide variability in dose, timing, regimens, and type of steroid given. A later UK survey by Allen et al. [64] demonstrated that 80% of the centres give steroids. According to a more recent US large database retrospective study in 2010, steroids were used in 54% of the paediatric cases. Despite wide use of glucocorticoids and proven anti-inflammatory effects in heart surgery with CPB [65–67], the majority of the studies have failed to show a survival benefit with steroids use [68–73]. Furthermore, large retrospective studies reported higher infection rates with steroids use [70, 71]. A recent large retrospective randomized trial in adult heart surgery found no significant effect of methylprednisolone on mortality or morbidity after cardiac surgery [74]. Similarly, a large, multicentre randomized trial in the paediatric population is warranted before firm recommendations can be made.

Steroids exert an anti-inflammatory effect by activating the cytoplasmic glucocorticoid receptor (GR) in the target tissues. The glucocorticoid-glucocorticoid receptor complex dissociates from its chaperone (the heat shock protein family hsp90) and translocates to the nucleus where it associates with the* glucocorticoid response elements* of various genes to induce expression of anti-inflammatory genes [75–77]. Steroids can also inhibit the activation of the NF-*κ*B redox sensitive transcription factors that play a central role in oxidative stress [76, 77].

Very few studies looked at the effect of steroids on inflammation and oxidative stress and cardiac function [76, 78]. Valen et al. [76] found increased activity of the tissue catalase and glutathione peroxidase in the isolated rat heart pretreated with methylprednisolone. Withington et al. [78] randomized 54 infants undergoing heart surgery with CPB to three different regimens of methylprednisolone (preoperative, at induction, and in the prime fluid). The prime administration group had less oxidative stress (higher glutathione to oxidised glutathione ratio).

Despite some studies [76] suggesting an increase of the antioxidant capacity with steroid treatment in the myocardium or other tissues [79], effects of glucocorticoids on oxidative stress remain controversial. For example, studies on hippocampal cell cultures demonstrated that steroids could promote oxidative stress induced death [80, 81]. Recently, several studies demonstrated a proinflammatory action of steroids depending on time and context of administration thus creating more controversy [82, 83].

### 4.3. Miniaturisation of the Extracorporeal Circuit

Mini-CPB bypass was developed in adult heart surgery in an attempt to reduce contact activation, air fluid interface, and cell damage by cardiotomy suction and haemodilution. Minimising such disadvantages of conventional CPB tempers the proinflammatory response to surgery but also the associated oxidative stress [84]. Adult studies demonstrated less oxidative stress with the use of mini-CPB [85]. The need to maintain an adequate haematocrit but also to overcome the patient's size and circuit discrepancy led to development of paediatric minicardiopulmonary. As seen earlier, the possibility of the bloodless prime eliminates the deleterious effects of blood transfusion. This is achieved in several ways: creation of biocompatible-coated circuits and oxygenators, vacuum assisted drainage systems, reducing the length of the circuit, or eliminating certain circuit components [84]. Miyaji et al. [86] successfully used mini-CPB in neonates weighing more than 4 kg. A more recent study by the same group demonstrated reduced inflammatory response with the use of mini-CPB in patients undergoing the Fontan procedure [87]. To our knowledge, there are no studies investigating differences between oxidative stress with conventional CPB and mini-CPB in paediatric heart surgery.

### 4.4. Cardioplegia, Pump Prime, and Oxidative Stress

Reactive oxygen species production can be controlled by enrichment of cardioplegia solution with antioxidants according to both experimental [88–90] and adult studies [91–95]. Early experimental studies demonstrated the superiority of blood cardioplegia over crystalloid cardioplegia in reducing oxidative damage [96]. This could be explained by the natural antioxidants blood constituents: haemoglobin, superoxide dismutase, catalase, and glutathione [90]. Despite these potential benefits of the blood cardioplegia, other studies suggested increased oxidative stress by either iron overload [40] or increase of hypoxanthine levels [97]. Finally, adult studies demonstrated less oxidative stress with intermittent antegrade warm blood cardioplegia compared to cold blood cardioplegia [98]. Calza et al. [23] found controlled antegrade low oxygen warm reperfusion to reduce oxidative stress and promote myocardial recovery.

Pump prime solutions used in neonatal heart surgery can also influence the antioxidant plasma capacity. This can prove to be significant because of the relation of the blood circulating volume and the volume of the extracorporeal circuit [99]. Previous animal studies demonstrated that supplementation of the prime solution with catalase reduces myocardial oxidative damage in the piglet heart [100]. Molicki et al. [99] investigated the antioxidant capacity of two different prime solutions for cardiopulmonary bypass in neonates: albumin or fresh frozen plasma based. Both prime solutions had no total radical antioxidant parameter value (ability of the investigated prime solution to inhibit peroxidation of a target lipid). However, the ferric-reducing ability (the capacity of the prime to reduce ferric ion to the ferrous form) was reduced in the prime solution compared to the undiluted, standard albumin and fresh frozen plasma. In their study, they also demonstrated that ultrafiltration led to loss of antioxidants and was ineffective in clearing lipid peroxidation products. In addition, adding mannitol to the prime had no antioxidant effect. The above study highlights not only the dilutional effect that results during prime preparation but also the depletion of antioxidants during ultrafiltration.

## 5. Conclusion

Cardiac surgery with the use of CPB is associated with altered redox states in children. Reactive oxygen species cause injury not only by both direct oxidation and peroxidation of cell membranes but also by cellular signalling. Contact with the paediatric bypass circuit surfaces activates the inflammatory cascade in which the neutrophil plays a central role in manufacturing of ROS. However, early inflammation-independent mechanisms such as haemolysis can also contribute to oxidative stress. The generation of ROS during ischaemic reperfusion injury can result in contractile dysfunction but nonreperfusion injury by reoxygenation of the chronically hypoxic heart can also occur. The lack of an antioxidant reserve or the coexistence of congenital heart defects associated with hypoxia or pressure load increases the vulnerability of the immature heart to oxidative damage. A multitude of interventions aimed at reducing ROS production or at increasing the antioxidant reserve were reviewed: exogenous antioxidants, steroids, miniaturisation of the CPB circuit, prime fluid, or cardioplegia strategies. The effect of such strategies on clinical outcomes, however, remains controversial. Further clinical studies looking at the effect of oxidative stress modulation on clinical outcomes after paediatric heart surgery are needed.

## References

[1] Desborough J. P. (2000). The stress response to trauma and surgery. *British Journal of Anaesthesia*.

[2] Laffey J. G., Boylan J. F., Cheng D. C. H. (2002). The systemic inflammatory response to cardiac surgery: implications for the anesthesiologist. *Anesthesiology*.

[3] Clermont G., Vergely C., Jazayeri S. (2002). Systemic free radical activation is a major event involved in myocardial oxidative stress related to cardiopulmonary bypass. *Anesthesiology*.

[4] Zakkar M., Guida G., Suleiman M.-S., Angelini G. D. (2015). Cardiopulmonary bypass and oxidative stress. *Oxidative Medicine and Cellular Longevity*.

[5] Babior B. M. (2000). Phagocytes and oxidative stress. *The American Journal of Medicine*.

[6] Kouchoukos N. T., Blackstone E. H., Hanley F. L., Kirklin J. K. (2012). *Kirklin/Barratt-Boyes Cardiac Surgery*.

[7] Kumar V. (2013). Cell injury, cell death, and adaptations. *Robbins Basic Pathology*.

[8] McDonald C. I., Fraser J. F., Coombes J. S., Fung Y. L. (2014). Oxidative stress during extracorporeal circulation. *European Journal of Cardio-Thoracic Surgery*.

[9] Ott M., Gogvadze V., Orrenius S., Zhivotovsky B. (2007). Mitochondria, oxidative stress and cell death. *Apoptosis*.

[10] Szeto H. H. (2008). Mitochondria-targeted cytoprotective peptides for ischemia-reperfusion injury. *Antioxidants and Redox Signaling*.

[11] Dai D. F., Hsieh E. J., Liu Y. (2012). Mitochondrial proteome remodelling in pressure overload-induced heart failure: the role of mitochondrial oxidative stress. *Cardiovascular Research*.

[12] Dare A. J., Logan A., Prime T. A. (2015). The mitochondria-targeted anti-oxidant MitoQ decreases ischemia-reperfusion injury in a murine syngeneic heart transplant model. *Journal of Heart and Lung Transplantation*.

[13] Oyewole A. O., Birch-Machin M. A. (2015). Mitochondria-targeted antioxidants. *The FASEB Journal*.

[14] Saugstad O. D. (2003). Bronchopulmonary dysplasia—oxidative stress and antioxidants. *Seminars in Neonatology*.

[15] Huang Q., Zhou H. J., Zhang H. (2015). Thioredoxin-2 inhibits mitochondrial reactive oxygen species generation and apoptosis stress kinase-1 activity to maintain cardiac function. *Circulation*.

[16] Manso P. H., Carmona F., Dal-Pizzol F. (2013). Oxidative stress markers are not associated with outcomes after pediatric heart surgery. *Paediatric Anaesthesia*.

[17] Gutierrez J., Ballinger S. W., Darley-Usmar V. M., Landar A. (2006). Free radicals, mitochondria, and oxidized lipids. The emerging role in signal transduction in vascular cells. *Circulation Research*.

[18] Morgan M. J., Liu Z. G. (2011). Crosstalk of reactive oxygen species and NF-kappaB signaling. *Cell Research*.

[19] Christen S., Finckh B., Lykkesfeldt J. (2005). Oxidative stress precedes peak systemic inflammatory response in pediatric patients undergoing cardiopulmonary bypass operation. *Free Radical Biology and Medicine*.

[20] Kawahito K., Kobayashi E., Ohmori M. (2000). Enhanced responsiveness of circulatory neutrophils after cardiopulmonary bypass: increased aggregability and superoxide producing capacity. *Artificial Organs*.

[21] Karu I., Taal G., Zilmer K., Pruunsild C., Starkopf J., Zilmer R. (2010). Inflammatory/oxidative stress during the first week after different types of cardiac surgery. *Scandinavian Cardiovascular Journal*.

[22] Schindhelm R. K., van der Zwan L. P., Teerlink T., Scheffer P. G. (2009). Myeloperoxidase: a useful biomarker for cardiovascular disease risk stratification?. *Clinical Chemistry*.

[23] Calza G., Lerzo F., Perfumo F. (2002). Clinical evaluation of oxidative stress and myocardial reperfusion injury in pediatric cardiac surgery. *Journal of Cardiovascular Surgery*.

[24] Gil-Gómez R., Blasco-Alonso J., Castillo-Martín R., Milano-Manso G. (2016). Indicadores pronósticos clínicos en el posoperatorio de cirugía cardiovascular pediátrica y su relación con la cinética del estrés oxidativo. *Revista Española de Anestesiología y Reanimación*.

[25] Caputo M., Mokhtari A., Miceli A. (2014). Controlled reoxygenation during cardiopulmonary bypass decreases markers of organ damage, inflammation, and oxidative stress in single-ventricle patients undergoing pediatric heart surgery. *Journal of Thoracic and Cardiovascular Surgery*.

[26] Caputo M., Mokhtari A., Rogers C. A. (2009). The effects of normoxic versus hyperoxic cardiopulmonary bypass on oxidative stress and inflammatory response in cyanotic pediatric patients undergoing open cardiac surgery: a randomized controlled trial. *The Journal of Thoracic and Cardiovascular Surgery*.

[27] Hirthler M., Simoni J., Dickson M. (1992). Elevated levels of endotoxin, oxygen-derived free radicals, and cytokines during extracorporeal membrane oxygenation. *Journal of Pediatric Surgery*.

[28] Lefer D. J., Granger D. N. (2000). Oxidative stress and cardiac disease. *American Journal of Medicine*.

[29] Buckberg G. D. (1995). Studies of hypoxemic/reoxygenation injury. I. Linkage between cardiac function and oxidant damage. *The Journal of Thoracic and Cardiovascular Surgery*.

[30] Taggart D. P., Hadjinikolas L., Hooper J. (1997). Effects of age and ischemic times on biochemical evidence of myocardial injury after pediatric cardiac operations. *The Journal of Thoracic and Cardiovascular Surgery*.

[31] Li R.-K., Mickle D. A. G., Weisel R. D. (1989). Effect of oxygen tension on the anti-oxidant enzyme activities of tetralogy of Fallot ventricular myocytes. *Journal of Molecular and Cellular Cardiology*.

[32] Morita K., Ihnken K., Buckberg G. D., Sherman M. P., Young H. H. (1995). Studies of hypoxemic/reoxygenation injury: without aortic clamping. IV. Role of the iron-catalyzed pathway: deferoxamine. *The Journal of Thoracic and Cardiovascular Surgery*.

[33] Cabigas E. B., Ding G., Chen T. (2012). Age- and chamber-specific differences in oxidative stress after ischemic injury. *Pediatric Cardiology*.

[34] Kloner R. A., Przyklenk K., Whittaker P. (1989). Deleterious effects of oxygen radicals in ischemia/reperfusion. Resolved and unresolved issues. *Circulation*.

[35] Oliveira M. S., Floriano E. M., Mazin S. C. (2011). Ischemic myocardial injuries after cardiac malformation repair in infants may be associated with oxidative stress mechanisms. *Cardiovascular Pathology*.

[36] Ihnken K., Morita K., Buckberg G. D. (1995). Studies of hypoxemic/reoxygenation injury: without aortic clamping. II. Evidence for reoxygenation damage. *The Journal of Thoracic and Cardiovascular Surgery*.

[37] Cavarocchi N. C., England M. D., O'Brien J. F. (1986). Superoxide generation during cardiopulmonary bypass: is there a role for vitamin E?. *Journal of Surgical Research*.

[38] Cavarocchi N. C., England M. D., Schaff H. V. (1986). Oxygen free radical generation during cardiopulmonary bypass: correlation with complement activation. *Circulation*.

[39] Pyles L. A., Fortney J. E., Kudlak J. J., Gustafson R. A., Einzig S. (1995). Plasma antioxidant depletion after cardiopulmonary bypass in operations for congenital heart disease. *The Journal of Thoracic and Cardiovascular Surgery*.

[40] Mumby S., Chaturvedi R. R., Brierley J. (2000). Iron overload in paediatrics undergoing cardiopulmonary bypass. *Biochimica et Biophysica Acta*.

[41] Wright G. (2001). Haemolysis during cardiopulmonary bypass: Update. *Perfusion*.

[42] Mamikonian L. S., Mamo L. B., Smith P. B., Koo J., Lodge A. J., Turi J. L. (2014). Cardiopulmonary bypass is associated with hemolysis and acute kidney injury in neonates, infants, and children^*^. *Pediatric Critical Care Medicine*.

[43] Mumby S., Koh T. W., Pepper J. R., Gutteridge J. M. C. (2001). Risk of iron overload is decreased in beating heart coronary artery surgery compared to conventional bypass. *Biochimica et Biophysica Acta*.

[44] Chaturvedi R. R., Shore D. F., Lincoln C. (1999). Acute right ventricular restrictive physiology after repair of tetralogy of fallot: association with myocardial injury and oxidative stress. *Circulation*.

[45] Simpson S. A., Zaccagni H., Bichell D. P. (2014). Acetaminophen attenuates lipid peroxidation in children undergoing cardiopulmonary bypass. *Pediatric Critical Care Medicine*.

[46] Gitto E., Karbownik M., Reiter R. J. (2001). Effects of melatonin treatment in septic newborns. *Pediatric Research*.

[47] Kiziltepe U., Tunçtan B., Eyileten Z. B. (2004). Efficiency of L-arginine enriched cardioplegia and non-cardioplegic reperfusion in ischemic hearts. *International Journal of Cardiology*.

[48] Ozaydin M., Peker O., Erdogan D. (2008). N-acetylcysteine for the prevention of postoperative atrial fibrillation: a prospective, randomized, placebo-controlled pilot study. *European Heart Journal*.

[49] Adabag A. S., Ishani A., Bloomfield H. E., Ngo A. K., Wilt T. J. (2009). Efficacy of N-acetylcysteine in preventing renal injury after heart surgery: a systematic review of randomized trials. *European Heart Journal*.

[50] England M. D., Cavarocchi N. C., O'Brien J. F. (1986). Influence of antioxidants (mannitol and allopurinol) on oxygen free radical generation during and after cardiopulmonary bypass. *Circulation*.

[51] Bochenek A., Religa Z., Spyt T. J. (1990). Protective influence of pretreatment with allopurinol on myocardial function in patients undergoing coronary artery surgery. *European Journal of Cardio-Thoracic Surgery*.

[52] Coetzee A., Roussouw G., Macgregor L. (1996). Failure of allopurinol to improve left ventricular stroke work after cardiopulmonary bypass surgery. *Journal of Cardiothoracic and Vascular Anesthesia*.

[53] Marro P. J., Baumgart S., Delivoria-Papadopoulos M. (1997). Purine metabolism and inhibition of xanthine oxidase in severely hypoxic neonates going onto extracorporeal membrane oxygenation. *Pediatric Research*.

[54] Xia W.-F., Liu Y., Zhou Q.-S., Tang Q.-Z., Zou H.-D. (2011). Protective effect of propofol and its relation to postoperation recovery in children undergoing cardiac surgery with cardiopulmonary bypass. *Pediatric Cardiology*.

[55] Xia W.-F., Liu Y., Zhou Q.-S., Tang Q.-Z., Zou H.-D. (2011). Comparison of the effects of propofol and midazolam on inflammation and oxidase stress in children with congenital heart disease undergoing cardiac surgery. *Yonsei Medical Journal*.

[56] Xia Z., Gu J., Ansley D. M., Xia F., Yu J. (2003). Antioxidant therapy with Salvia miltiorrhiza decreases plasma endothelin-1 and thromboxane B2 after cardiopulmonary bypass in patients with congenital heart disease. *Journal of Thoracic and Cardiovascular Surgery*.

[57] Gitto E., Romeo C., Reiter R. J. (2004). Melatonin reduces oxidative stress in surgical neonates. *Journal of Pediatric Surgery*.

[58] Reiter R. J., Calvo J. R., Karbownik M., Qi W., Tan D. X. (2000). Melatonin and its relation to the immune system and inflammation. *Annals of the New York Academy of Sciences*.

[59] Yang Y., Sun Y., Yi W. (2014). A review of melatonin as a suitable antioxidant against myocardial ischemia-reperfusion injury and clinical heart diseases. *Journal of Pineal Research*.

[60] Broche F., Romero A., Olembe E. (2002). Aprotinin mediated antioxidant effect in cardiosurgery with mechanical cardiorespiratory support (CMCS). *Journal of Cardiovascular Surgery*.

[61] Broche V. F., Romero Suàrez A., Olembe E. (1996). Aprotinin effects related to oxidative stress in cardiosurgery with mechanical cardiorespiratory support (CMCS). *Annals of the New York Academy of Sciences*.

[62] Adlam V. J., Harrison J. C., Porteous C. M. (2005). Targeting an antioxidant to mitochondria decreases cardiac ischemia-reperfusion injury. *The FASEB Journal*.

[63] Checchia P. A., Bronicki R. A., Costello J. M., Nelson D. P. (2005). Steroid use before pediatric cardiac operations using cardiopulmonary bypass: an international survey of 36 centers. *Pediatric Critical Care Medicine*.

[64] Allen M., Sundararajan S., Pathan N., Burmester M., MacRae D. (2009). Anti-inflammatory modalities: their current use in pediatric cardiac surgery in the United Kingdom and Ireland. *Pediatric Critical Care Medicine*.

[65] Bronicki R. A., Backer C. L., Baden H. P., Mavroudis C., Crawford S. E., Green T. P. (2000). Dexamethasone reduces the inflammatory response to cardiopulmonary bypass in children. *Annals of Thoracic Surgery*.

[66] Lindberg L., Forsell C., Jögi P., Olsson A.-K. (2003). Effects of dexamethasone on clinical course, C-reactive protein, S100B protein and von Willebrand factor antigen after paediatric cardiac surgery. *British Journal of Anaesthesia*.

[67] Ando M., Park I.-S., Wada N., Takahashi Y. (2005). Steroid supplementation: a legitimate pharmacotherapy after neonatal open heart surgery. *Annals of Thoracic Surgery*.

[68] Dreher M., Glatz A. C., Kennedy A., Rosenthal T., Gaynor J. W. (2015). A single-center analysis of methylprednisolone use during pediatric cardiopulmonary bypass. *The Journal of Extra-Corporeal Technology*.

[69] Keski-Nisula J., Pesonen E., Olkkola K. T. (2013). Methylprednisolone in neonatal cardiac surgery: reduced inflammation without improved clinical outcome. *The Annals of Thoracic Surgery*.

[70] Pasquali S. K., Hall M., Li J. S. (2010). Corticosteroids and outcome in children undergoing congenital heart surgery: analysis of the pediatric health information systems database. *Circulation*.

[71] Pasquali S. K., Li J. S., He X. (2012). Perioperative methylprednisolone and outcome in neonates undergoing heart surgery. *Pediatrics*.

[72] Robertson-Malt S., Afrane B., El Barbary M. (2007). Prophylactic steroids for pediatric open heart surgery. *Cochrane Database of Systematic Reviews*.

[73] Robertson-Malt S., El Barbary M. (2008). Prophylactic steroids for paediatric open-heart surgery: a systematic review. *International Journal of Evidence-Based Healthcare*.

[74] Whitlock R. P., Devereaux P. J., Teoh K. H. (2015). Methylprednisolone in patients undergoing cardiopulmonary bypass (SIRS): a randomised, double-blind, placebo-controlled trial. *The Lancet*.

[75] Ito K., Chung K. F., Adcock I. M. (2006). Update on glucocorticoid action and resistance. *Journal of Allergy and Clinical Immunology*.

[76] Valen G., Kawakami T., Tähepôld P. (2000). Pretreatment with methylprednisolone protects the isolated rat heart against ischaemic and oxidative damage. *Free Radical Research*.

[77] Adcock I. M., Barnes P. J. (2008). Molecular mechanisms of corticosteroid resistance. *Chest*.

[78] Withington D. E., Fontela P. S., Harrington K. P., Tchervenkov C., Lands L. C. (2014). A comparison of three dose timings of methylprednisolone in infant cardiopulmonary bypass. *SpringerPlus*.

[79] Costantini D., Marasco V., Møller A. P., Costantini D. (2011). A meta-analysis of glucocorticoids as modulators of oxidative stress in vertebrates. *Journal of Comparative Physiology B*.

[80] You J.-M., Yun S., Nam K. N., Kang C., Won R., Lee E. H. (2009). Mechanism of glucocorticoid-induced oxidative stress in rat hippocampal slice cultures. *Canadian Journal of Physiology and Pharmacology*.

[81] Behl C., Lezoualc'h F., Trapp T., Widmann M., Skutella T., Holsboer F. (1997). Glucocorticoids enhance oxidative stress-induced cell death in hippocampal neurons in vitro. *Endocrinology*.

[82] Yeager M. P., Pioli P. A., Guyre P. M. (2011). Cortisol exerts bi-phasic regulation of inflammation in humans. *Dose-Response*.

[83] Yeager M. P., Rassias A. J., Pioli P. A. (2009). Pretreatment with stress cortisol enhances the human systemic inflammatory response to bacterial endotoxin. *Critical Care Medicine*.

[84] Berkowitz D. H., Gaynor J. W., Mavroudis C., Backer C., Idriss R. F. (2013). Management of pediatric cardiopulmonary bypass. *Pediatric Cardiac Surgery*.

[85] van Boven W.-J., Gerritsen W. B., Waanders F. G., Haas F. J., Aarts L. P. (2004). Mini extracorporeal circuit for coronary artery bypass grafting: initial clinical and biochemical results: a comparison with conventional and off-pump coronary artery bypass grafts concerning global oxidative stress and alveolar function. *Perfusion*.

[86] Miyaji K., Miyamoto T., Kohira S. (2008). Miniaturized cardiopulmonary bypass system in neonates and small infants. *Interactive Cardiovascular and Thoracic Surgery*.

[87] Itatani K., Miyaji K., Miyamoto T. (2010). Miniaturized biocompatible cardiopulmonary bypass for the Fontan procedure. *Surgery Today*.

[88] Chambers D. J., Astras G., Takahashi A., Manning A. S., Braimbridge M. V., Hearse D. J. (1989). Free radicals and cardioplegia: organic anti-oxidants as additives to the St Thomas' hospital cardioplegic solution. *Cardiovascular Research*.

[89] Greenfield D. T., Greenfield L. J., Hess M. L. (1988). Enhancement of crystalloid cardioplegic protection against global normothermic ischemia by superoxide dismutase plus catalase but not diltiazem in the isolated, working rat heart. *The Journal of Thoracic and Cardiovascular Surgery*.

[90] Rosenkranz E. R. (1995). Substrate enhancement of cardioplegic solution: experimental studies and clinical evaluation. *The Annals of Thoracic Surgery*.

[91] Vento A., Nemlander A., Aittomäki J., Salo J., Karhunen J., Rämö O. J. (2003). N-Acetylcysteine as an additive to crystalloid cardioplegia increased oxidative stress capacity in CABG patients. *Scandinavian Cardiovascular Journal*.

[92] Ferreira R., Burgos M., Llesuy S. (1989). Reduction of reperfusion injury with mannitol cardioplegia. *The Annals of Thoracic Surgery*.

[93] Larsen M., Webb G., Kennington S. (2002). Mannitol in cardioplegia as an oxygen free radical scavenger measured by malondialdehyde. *Perfusion*.

[94] Suleiman M.-S., Fernando H. C., Dihmis W. C., Hutter J. A., Chapman R. A. (1993). A loss of taurine and other amino acids from ventricles of patients undergoing bypass surgery. *Heart*.

[95] Andrews D. T., Sutherland J., Dawson P., Royse A. G., Royse C. F. (2012). L-arginine cardioplegia reduces oxidative stress and preserves diastolic function in patients with low ejection fraction undergoing coronary artery surgery. *Anaesthesia and Intensive Care*.

[96] Julia P., Young H. H., Buckberg G. D., Kofsky E. R., Bugyi H. I. (1991). Studies of myocardial protection in the immature heart. IV. Improved tolerance of immature myocardium to hypoxia and ischemia by intravenous metabolic support. *Journal of Thoracic and Cardiovascular Surgery*.

[97] Quinlan G. J., Westerman S. T., Mumby S., Pepper J. R., Gutteridge J. M. C. (1999). Plasma hypoxanthine levels during crystalloid and blood cardioplegias: warm blood cardioplegia increases hypoxanthine levels with a greater risk of oxidative stress. *Journal of Cardiovascular Surgery*.

[98] Mezzetti A., Calafiore A. M., Lapenna D. (1995). Intermittent antegrade warm cardioplegia reduces oxidative stress and improves metabolism of the ischemic-reperfused human myocardium. *The Journal of Thoracic and Cardiovascular Surgery*.

[99] Molicki J. S., Draaisma A. M., Verbeet N. (2001). Prime solutions for cardiopulmonary bypass in neonates: antioxidant capacity of prime based on albumin or fresh frozen plasma. *Journal of Thoracic and Cardiovascular Surgery*.

[100] Ihnken K., Morita K., Buckberg G. D., Ihnken O., Winkelmann B., Sherman M. (1997). Prevention of reoxygenation injury in hypoxaemic immature hearts by priming the extracorporeal circuit with antioxidants. *Cardiovascular Surgery*.

